# Dentinogenesis imperfecta in a 6-year-old male neutered Labrador retriever: Case report with atypical clinical presentation and treatment review

**DOI:** 10.3389/fvets.2024.1473390

**Published:** 2024-11-04

**Authors:** Karolina Maria Piekos, Alix Freeman, Kathryn Fleming, Cynthia Bell

**Affiliations:** ^1^Department of Dentistry, The Ralph Veterinary Referral Centre, Marlow, United Kingdom; ^2^Department of Dentistry, Oral and Maxillofacial Surgery, Eastcott Veterinary Referrals, Part of Linnaeus Veterinary Limited, Swindon, United Kingdom; ^3^Department of Diagnostic Imaging, Anderson Moores Veterinary Specialists, Part of Linnaeus Veterinary Limited, Winchester, United Kingdom; ^4^Specialty Oral Pathology for Animals, LLC., Geneseo, IL, United States

**Keywords:** dentinogenesis imperfecta, dentine, dog, canine, enamel, abrasion, translucent teeth, discolouration

## Abstract

This case report details the diagnosis and treatment of dentinogenesis imperfecta in a 6-year-old neutered male Labrador, presenting without concurrent osteogenesis imperfecta. Diagnostic modalities, including radiographs, CT imaging, and histopathological examination, are reviewed in conjunction with the latest literature on canine dentinogenesis imperfecta. This patient presented at a more advanced age than typically reported cases. The clinical history, as provided by referring veterinarians, documented fractured deciduous teeth with delayed exfoliation. By 10 months of age, the patient’s permanent dentition exhibited a translucent appearance and structural anomalies. Upon presentation to Eastcott Referrals the patient was experiencing significant oral pain and exhibited generalised coronal wear with yellow/brown intrinsic discolouration. CT imaging revealed that all teeth had endodontic disease and associated apical periodontitis, with varied root canal widths indicating that teeth succumbed to endodontic disease at different time points. The treatment protocol involved staged full-mouth extractions, resulting in the complete resolution of clinical symptoms. This case underscores the importance of early diagnosis and intervention in managing dentinogenesis imperfecta in dogs.

## 1 Introduction

Dentinogenesis imperfecta (DGI) is an autosomal-dominant hereditary disorder in humans, characterised by defective dentine formation affecting both deciduous and permanent dentition. Dental abnormalities include generalised discolouration (commonly amber-brown), increased tooth fragility, pathological crown/root fractures, and significant dental abrasion (1). DGI is classified into three subtypes: Type I (DGI-I), which occurs in conjunction with osteogenesis imperfecta (OI), and types II and III (DGI-II, DGI-III), which are non-syndromic and limited to dental symptoms. In DGI, enamel can be easily exfoliated from the tooth’s surface due to abnormalities of the dentino-enamel junction (DEJ) or/and enamel hypomineralisation. This exposes the softer dentine underneath, leading to accelerated dental abrasion (1–4).

According to Shield’s dental phenotypic classification, three subtypes of DGI and two subtypes of dentine dysplasia (DD) type I (DD-I) and type II (DD-II) are recognised within the category of primary dentine disorders. DGI-II is considered milder than type III, while DGI-I is a dental manifestation of OI, mainly arising from mutations in the COL1A1 or COL1A2 genes (5, 6). Recent genetic research indicates that DGI-II, DGI-III, and DD-II are caused by mutations in the dentine sialophosphoprotein (DSPP) gene (7, 8). Significant phenotypic variation in individuals with DSPP mutations suggests that DD-II and DGI-II are different manifestations of the same genetic condition rather than separate diseases (8, 9). DD-I differs from the rest of primary dentine disorders in both genetic origin and clinical presentation. It is associated with rootless teeth and pulpal obliteration (5, 10). In humans, DD-I has been reported to follow an autosomal dominant pattern of inheritance and has recently been identified as a genetically heterogeneous condition linked to mutations in genes such as VPS4B, SSUH2, and SMOC2 (5, 11).

Adapting the human classification of primary dentine disorders to veterinary dentistry has been proposed due to the rarity and limited research of this disorder in dogs. Only four cases of non-syndromic DGI resembling human DGI-II have been reported in dogs, including this case, all treated with dental extractions due to severe symptoms and poor prognosis (12, 13).

Given the limited number of cases (12, 13), it remains uncertain whether Shield’s classification, widely used in human dentine disorders, can be directly applied to canine cases, especially in distinguishing between non-syndromic DGI subtypes. In humans, DGI-II is considered a milder form, while DGI-III is seen as a more severe variation (14). However, it is unclear if this distinction is applicable to dogs. We have chosen to adhere to this classification where applicable to avoid confusion with syndromic DGI (DGI-I) associated with OI.

Despite advancements in human genetics, Shield’s classification remains practical for diagnosing dentine disorders, providing a clear and accessible method for categorisation. In humans, genetic testing aids in determining the correct diagnosis, treatment and prognosis for DGI-II, DGI-III, and DD-II. In veterinary practice, the only available genetic test is for OI-related mutations in the COL1A1, COL1A2 and SERPINH1 genes.^1^ Diagnosis relies on a detailed history, pedigree analysis, and clinical examination, including radiographic assessment. The histological characteristics of teeth in OI are identical to those in DGI, making differentiation between them impossible; however, it helps distinguish dentine disorders from enamel disorders (15).

This case, diagnosed later in life, exhibited features of a milder form of DGI-II according to Shield’s classification. Early identification of this disease could facilitate conservative dental management strategies, whereas a late diagnosis is more likely to necessitate either selective or full-mouth extractions. This report reviews primary dentine disorder classification, differential diagnosis considerations, and human dental treatment options that may apply to less severely affected cases, for dedicated owners willing to pursue referral dental treatment.

## 2 Case description

A 6-year-old, male neutered Labrador Retriever was presented to a referral practice for evaluation of extrinsically stained permanent dentition and inappropriate abrasion. The puppy was acquired at 8 weeks of age, from a litter of 10. Initial physical examination, by the primary care veterinarian, conducted soon after purchase revealed no abnormalities in dentition or overall physical health. At 7 months of age, delayed exfoliation of the deciduous dentition and a complicated crown fracture of one of the deciduous canines was observed. Extraction was recommended. By the age of 10 months, complete exfoliation of the deciduous teeth was confirmed, accompanied by an abnormal translucency of the permanent dentition. The puppy continued to develop normally in all other aspects and was presented for further vaccinations at 15 months of age, with significant dental abrasion in addition to the previous findings. A detailed examination under general anaesthesia with full mouth dental radiography was recommended. In the absence of visible oral discomfort, the owner elected not to pursue further treatment, and continued to present the patient to the referring vets at 6-monthly intervals to monitor for pulp exposure. Additionally, a soft food diet was introduced, and hard chews and toys were discouraged.

The abrasion worsened, and the patient was reported to have developed signs of oral discomfort at 5 years of age including pain during mastication of hard food, a preference for a soft diet, reluctance to drink water, and withdrawal from usual activities and playful behaviours with other dogs. Oral examination in the conscious patient revealed significant loss of tooth substance with generalised discolouration of the teeth. Referral to a dentistry and oral surgery service was recommended.

## 3 Clinical findings

At presentation to the referral dentistry service, the patient was in good body condition, weighed 24.5 kg, with an unremarkable general physical examination. Oral examination revealed severe abrasion affecting all teeth. Exposed dentine exhibited amber-brown discolouration. Some teeth were worn to the level of the gingival margin. Moderate calculus accumulation was present with no signs of periodontitis. The patient had a missing left mandibular second incisor (302) and right mandibular first incisor (401), with otherwise complete adult dentition and normocclusion.

## 4 Treatment and diagnostic investigation

A detailed evaluation of the oral cavity under general anaesthesia, utilising computed tomography (CT) and intra-oral dental radiography, was recommended to assess tooth vitality.

### 4.1 Oral examination findings under general anaesthesia

Intravenous premedication with medetomidine^2^ (0.005 mg/kg) and methadone^3^ (0.2 mg/kg) was administered, followed by propofol^4^ (4 mg/kg) intravenously to induce general anaesthesia. Anaesthesia was maintained with isoflurane.^5^ Intraoperative medications included intravenous methadone 0.1 mg/kg, medetomidine(0.0015 mg/kg), acepromazine^6^ (0.001 mg/kg), paracetamol^7^ (10 mg/kg) and meloxicam^8^ (0.2 mg/kg) along with a ketamine^9^ constant rate infusion administered at 2.5-10mcg/kg/min.

**Figure 1:**
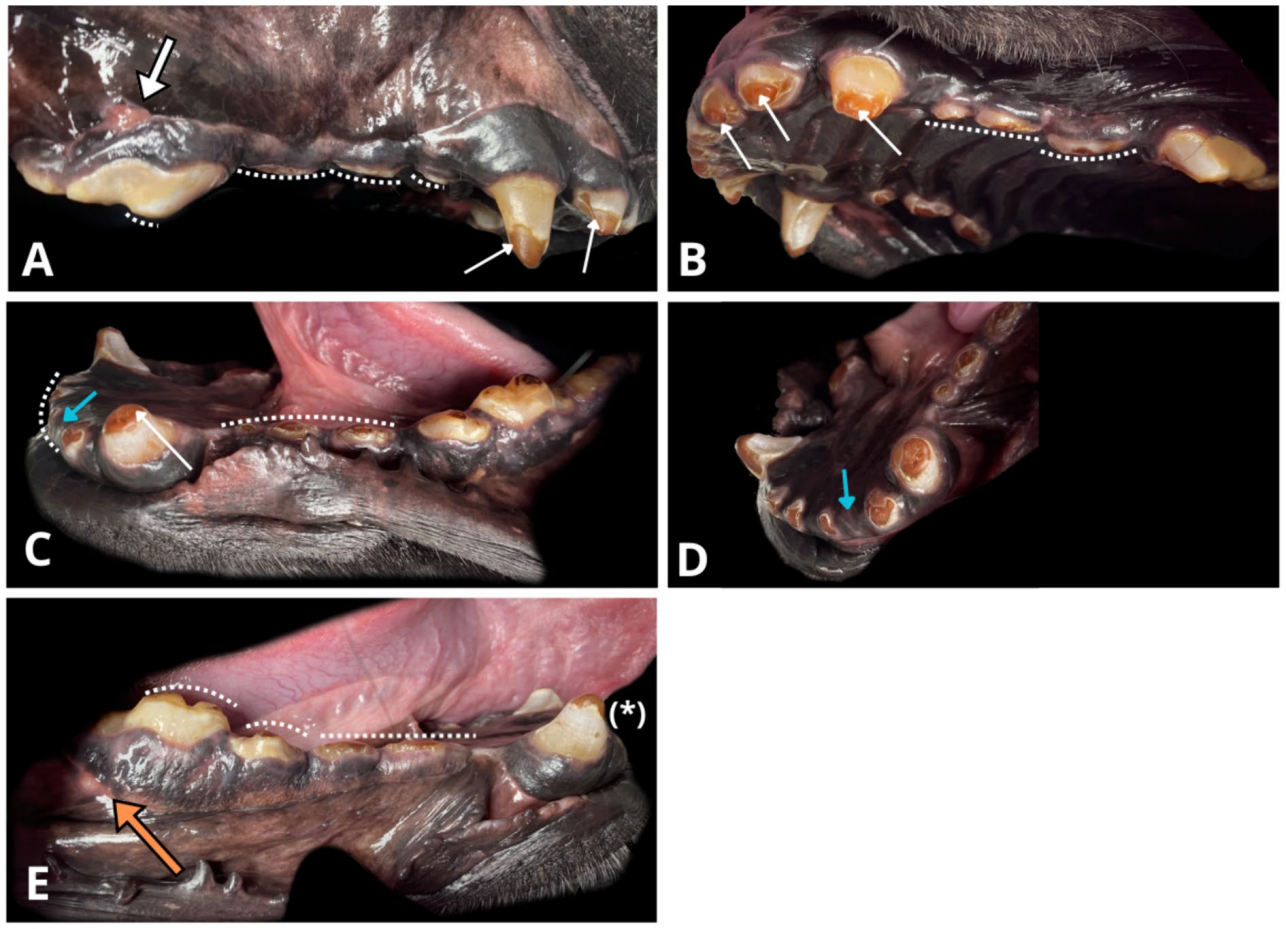
Photographs of the oral cavity obtained with the patient under general anaesthesia. Note the amber-brown discolouration of exposed dentine and the grey-brown appearance of the remaining enamel (white narrow arrows). Visible generalised severe crown loss due to abnormal abrasion (white dotted line) and enamel thinning over occlusal surfaces. **(A)** Right maxillary view, draining tract on the mucogingival junction (thick white arrow) associated with the radiographically evident periapical lucency around the distal root of the right maxillary fourth premolar (108). **(B)** Left maxillary and palatal view. **(C)** Left mandibular view. Crown fractures at the level of cemento-enamel junction of the left mandibular second incisor 302 (blue arrow), right mandibular first incisor (401), with their roots *in situ*. **(D)** Rostral left mandibular view. Another angle on missing left mandibular second incisor (302, blue arrow) with radiographically confirmed retained root covered by healthy gingiva. **(E)** Right mandibular view. Enamel infractions on the right mandibular canine (404, asterisk). A visible draining tract (thick, orange arrow) associated with radiographic periapical hypoattenuation at the distal root of the right mandibular first molar (409).

An oral examination under general anaesthesia (Figure 1) showed two sinus tracts associated with the right maxillary fourth premolar (108) and the right mandibular first molar (409) teeth. Mild gingivitis was evident, with no abnormal periodontal probing depths or other pathology affecting the soft tissues.

Areas of significant abrasion on the occlusal surfaces of the teeth, where crown tooth matter was lost to the level of the gingival margin, were present on all remaining mandibular incisors (301, 303, 402, 403), 11 out of 16 premolars including the first, second, and third maxillary premolars bilaterally (105, 106, 107, 205, 206, 207), as well as the first and second mandibular premolars bilaterally (305, 306, 405, 406) along with the third mandibular premolar on the left (307). The crowns of the remaining dentition were also abraded, to a lesser extent.

The enamel distribution across all dentition was limited, extending only over the cervical and middle portions of the remaining crowns, with no presence on the occlusal surfaces. Enamel infractions were identified on the right mandibular canine (404) and right first mandibular molar (409). The exposed dentine exhibited a widespread intrinsic amber-brown discolouration. The enamel had a grey-brown hue.

### 4.2 Diagnostic imaging findings

**Figure 2:**
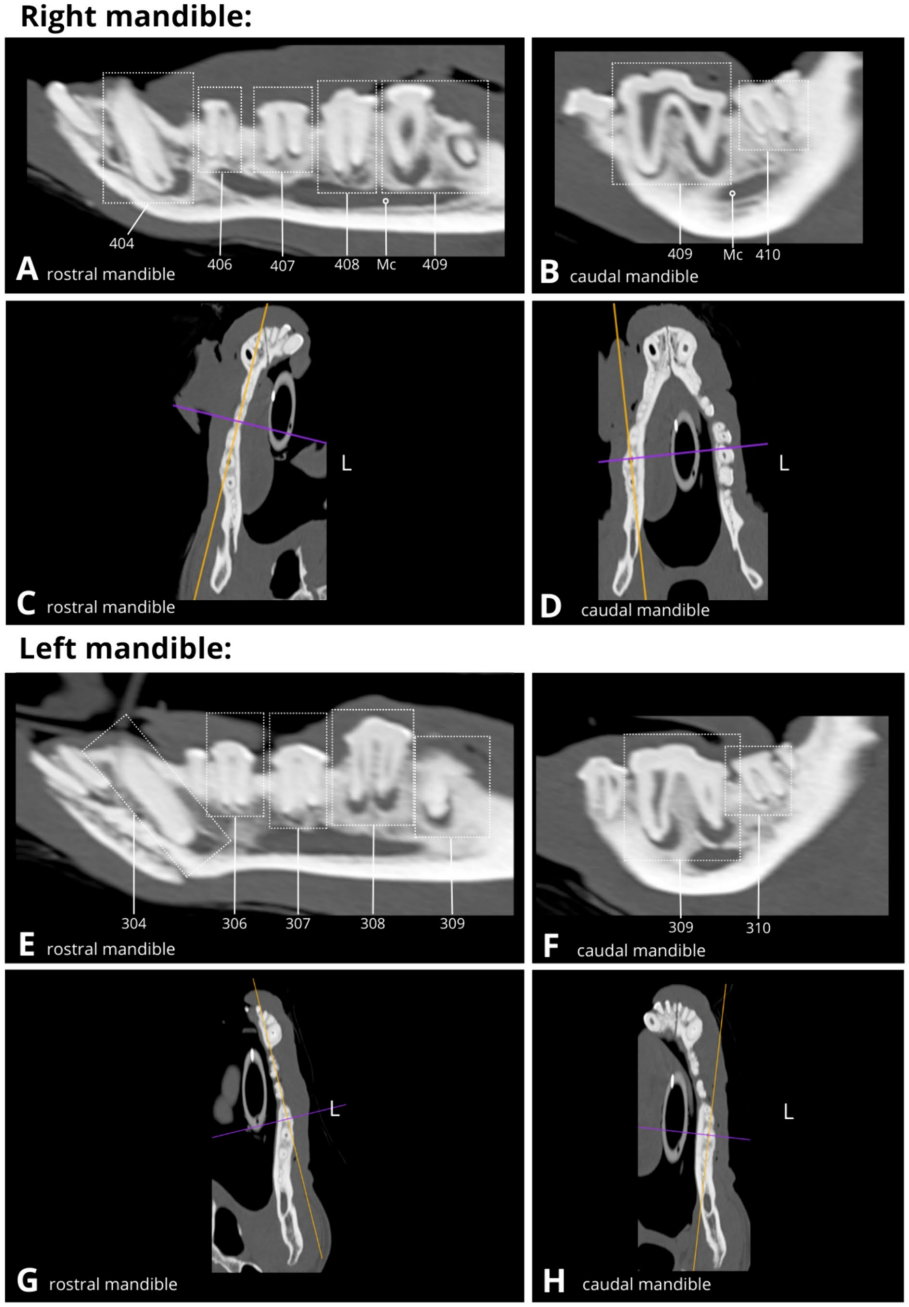
Slightly oblique sagittal **(A,B,E,F)** and dorsal **(B,C,G,H)** CT reconstructions display the dentition (dotted rectangles) of the right and left mandible, demonstrating multiple variable-sized areas of periapical hypoattenuation consistent with apical periodontitis (see **A**,**B**,**E**,**F**). Mandibular canal (Mc), Left (L).

High resolution head CT with intravenous contrast showed periapical hypoattenuation of variable severity across all teeth, suggestive of apical periodontitis (Figure 2). The CT exhibited greater sensitivity in detecting periapical radiolucency compared to intraoral radiographic findings, with the pathology identified in only eight teeth using the latter modality. On the CT scan, the hypoattenuating areas were largest at the apices of all four canine teeth (104, 204, 304, 404), the left maxillary third premolar (207), the right and left maxillary fourth premolars (108, 208), the left mandibular fourth premolar (308) and both right and left mandibular first molars (409, 309).

Furthermore, multiple areas of sclerosis within the alveolar bone were evident, surrounding the peripheries of the periapical hypoattenuation, consistent with condensing periostitis, most pronounced in the right rostral mandible associated with the right mandibular canine tooth (404), and comparatively less apparent in proximity to the right maxillary fourth premolar (108), the left maxillary third and fourth premolars (207 and 208), the left mandibular fourth premolar (308), the left mandibular first molar (309), and the right mandibular third premolar and first molar (407 and 409).

Additionally, small areas of focal buccal cortical lysis were identified, suggesting possible draining tracts, all confluent with periapical hypoattenuation linked to the distal root of the right maxillary fourth premolar (108), the distal root of the right mandibular first molar (409), and partially over the distal root of the left maxillary third premolar (207). Adjacent to these teeth, there was a very mild, irregular periosteal reaction observed over the buccal aspects of the mandible and maxilla, and additionally lateral to the left mandibular first molar (309), indicative of proliferative periostitis or osteomyelitis.

Overall, tooth root length and shape appeared normal, and all dentition exhibited closed apices. Full assessment of the crown morphology was limited due to the significant loss of dental hard tissue. Crown fractures were identified in three instances: at the tip of the crown of the right mandibular first premolar (405) and at the level of the cementoenamel junction (CEJ) of the right mandibular first incisor and left mandibular second incisor (401 and 302) with their roots *in situ*.

**Figure 3:**
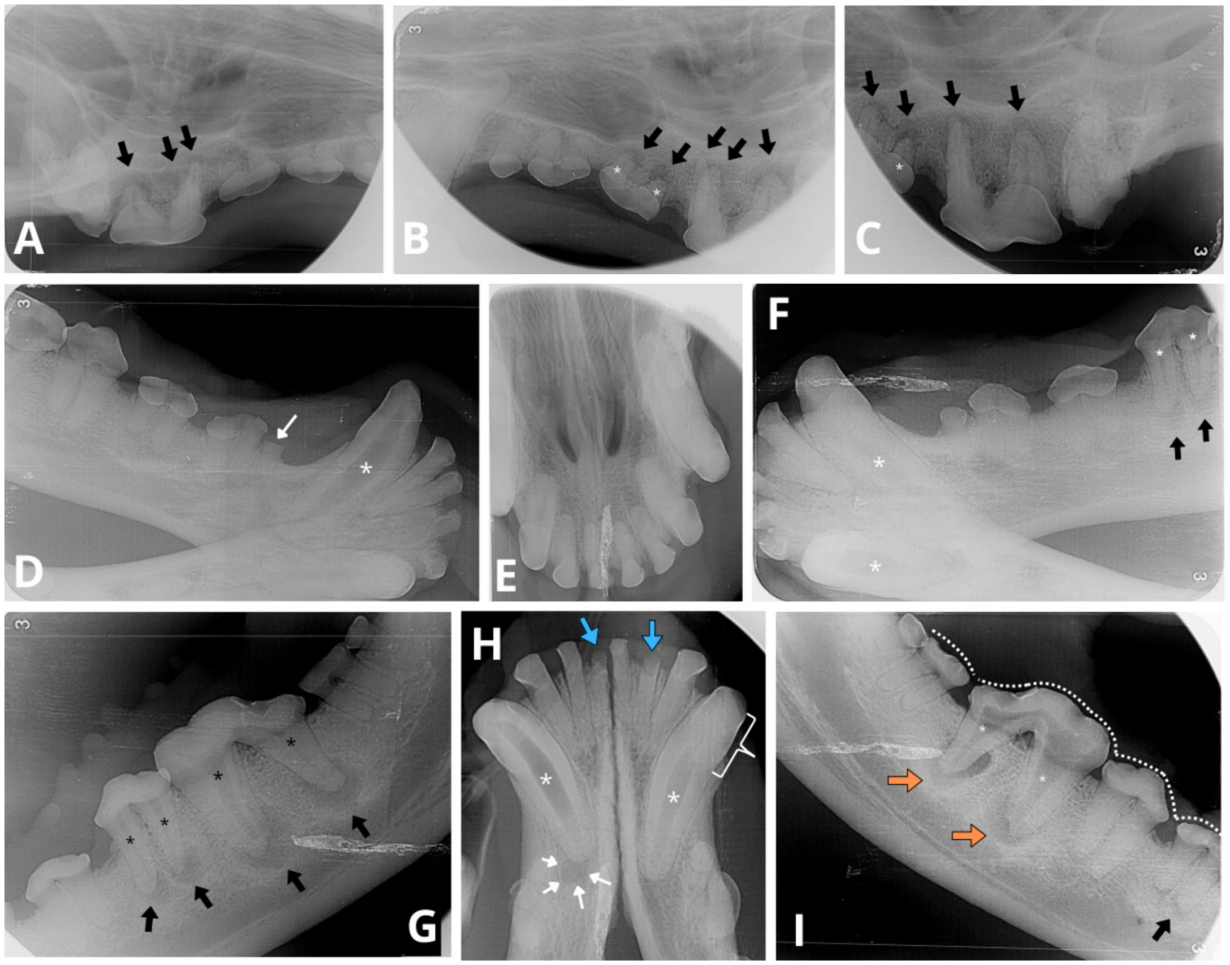
Intraoral radiographs reveal indications of apical periodontitis, variable widths of the root canals and abnormal tooth wear. **(A)** Lateral view of the right maxillary premolar and molar teeth displays periapical lucency of the bone surrounding each root apex of the fourth right maxillary premolar 108 (black arrows). **(B,C)** Lateral views of the left maxillary premolar and molar teeth depict similar periapical bone loss observed on all roots of left maxillary third and fourth premolar (207 and 208, respectively, black arrows). Abnormally wide root canals (white asterisk) are noticeable in the left maxillary third premolar (207) compared to the adjacent tooth—left maxillary second premolar (206). **(D)** Lateral view of the right mandibular canine and premolar teeth reveals a fracture above the alveolar bone line crown (white thin arrow) of the first right mandibular premolar (405). An abnormally wide and radiolucent pulp cavity and root canal are observed in the right mandibular canine (404; white asterisk). **(E)** Occlusal view of the maxillary incisors and canine teeth shows no evident signs of periapical pathology or abnormal width of the root canals. **(F)** Lateral view of the left mandibular canine (304) and premolar teeth displays evident signs of periapical pathology associated with the mesial and distal roots of the left mandibular fourth premolar (black arrows). Evidence of endodontic disease is depicted by abnormally wide pulp cavity and root canals in the right and left mandibular canines (404, 304) and the left mandibular fourth premolar (308; white asterisks). **(G)** Lateral view of the left mandible indicates periapical lucency in all root apices of the left mandibular fourth premolar (308) and the left mandibular first molar (309; black arrows). Abnormally wide root canals are also evident in both instances (black asterisk). A relative difference in root canal width is observed between the left mandibular fourth premolar (308) and its counterpart, the right mandibular fourth premolar (408, radiograph **I**), where in the latter both root canals have a normal width for the dog’s age. **(H)** The occlusal view of the mandibular incisor and canine teeth reveals periapical lucency of the bone around the apex of the right mandibular canine (404, white thin arrows). Both mandibular canines exhibit visibly abnormally wide root canals (white asterisks). The pulp chamber of the right mandibular canine (404) is more radiolucent compared to the left mandibular canine (304), consistent with the presence of gas content within the pulp chamber. Additionally, the left mandibular canine (304) displays partial obliteration of the pulp chamber, manifesting as tapered narrowing in its coronal aspect (white bracket), resembling a flame-shaped cavity. Furthermore, two mandibular incisors, the left mandibular second incisor (302) and the right mandibular first incisor (401), exhibit visible crown fractures (blue thick arrows), with retention of their roots. The periodontal ligament space surrounding the apical portion of the incisor roots is poorly discernible, indicative of external replacement resorption. **(I)** Lateral view of the right mandible shows majority of teeth cusps to be blunted (white dotted line) as observed in other views. The right mandibular first molar (409) presents evident signs of endodontic disease with bone loss around the resorbing roots (undergoing external inflammatory resorption), and both root canals are similarly abnormally wide (white asterisk). Clearly circumscribed periapical lucent areas are visible in both distal and mesial roots (orange arrows). Thinner dentine walls of the right mandibular first molar (409) compared to its counterpart the left maxillary first molar (309, see radiograph **G**) suggest an earlier arrested maturation of tooth 409. External inflammatory resorption is indicated by the irregular shape of the apical portion of the roots in both 409 and 309 (see radiograph **G**). Additionally, the mesial root of right mandibular third premolar (407) shows signs of mild periapical lucency (black arrow).

**Figure 4:**
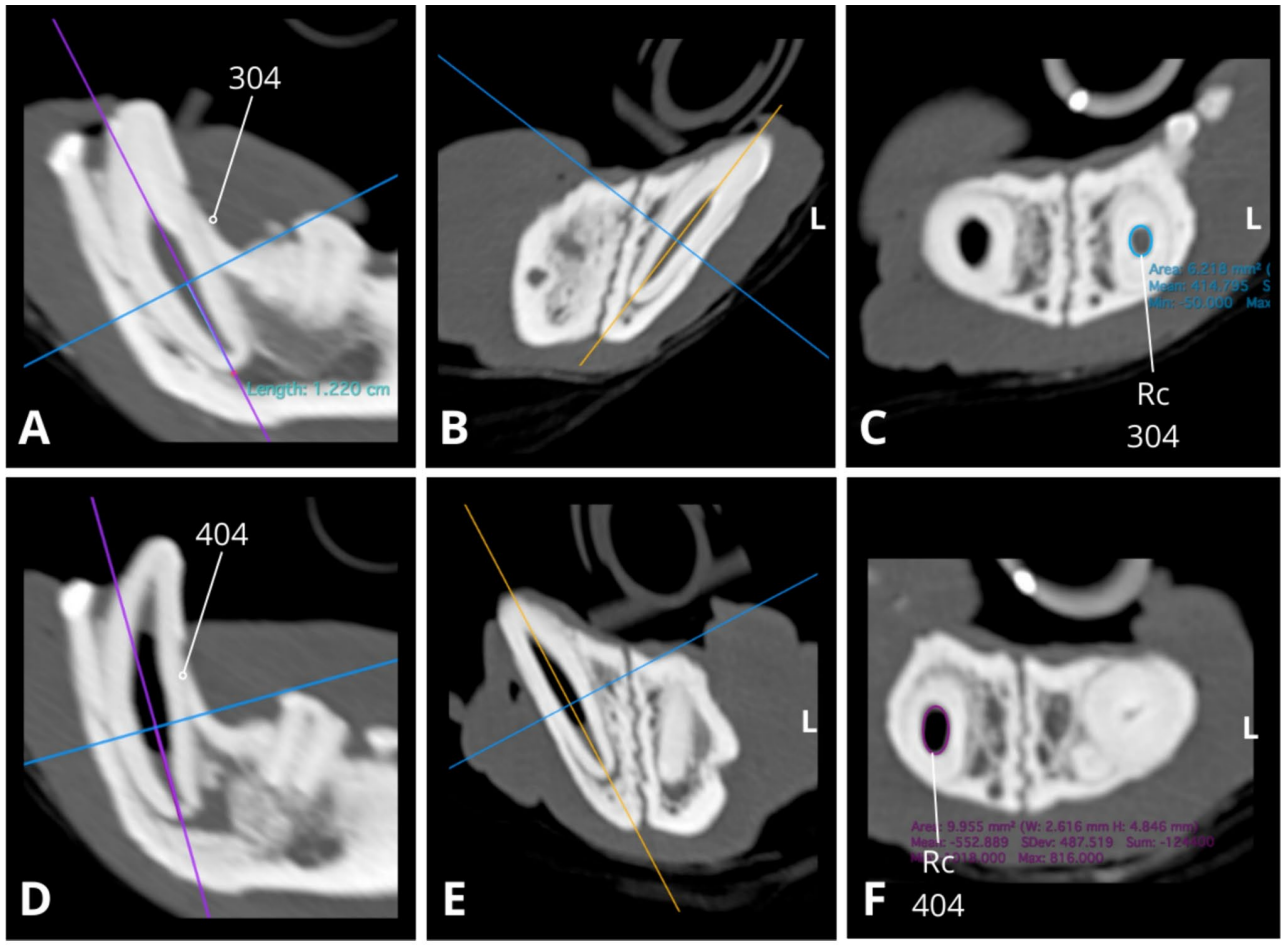
Slightly oblique sagittal **(A,D)**, transverse **(B,E)**, and dorsal **(C,F)** CT reconstructions illustrate relative differences in root canal width between the mandibular canines (304, 404), with the right mandibular canine canal notably wider **(D,F)**. Root canal cross-section (Rc) measurements were conducted at a consistent level along the long axis of the root (1.22 cm from the apex border) with slightly oblique dorsal CT reconstructions **(C,F)** used to obtain transverse images of the root canal. The approximate cross-sectional area of the root canal for the left mandibular canine 304 is 6.218 mm^2^ (**C**, blue circle; dimensions: 2.4 × 3.3 mm), while for the right mandibular canine 404, it measures 10.0 mm^2^ (**F**, purple circle; dimensions: 2.6 × 4.8 mm). Measures obtained using Horos, open-source medical image viewer (RRID:SCR_017340).

**Figure 5 fig5:**
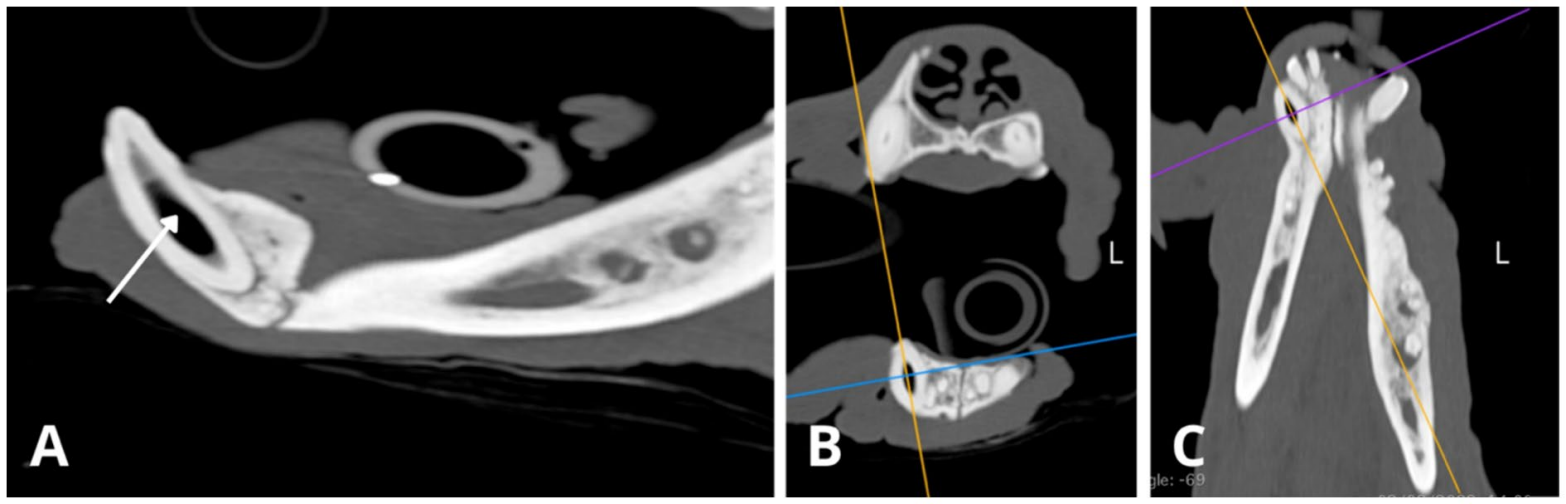
Slightly oblique sagittal **(A)**, transverse **(B)**, and dorsal **(C)** CT reconstructions illustrate the right mandibular canine (404). There is an abnormally wide pulp cavity, along with gas accumulation within the central portion of the cavity (indicated by the white arrow).

**Figure 6:**
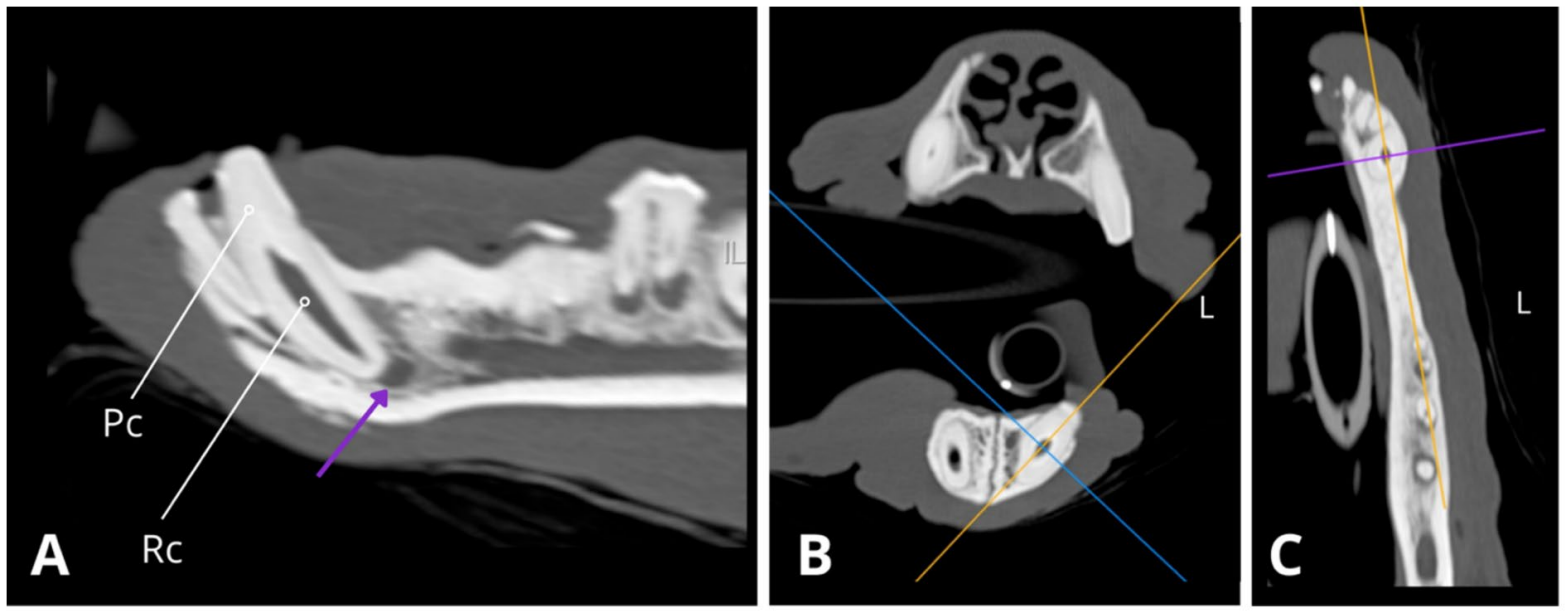
Slightly oblique sagittal **(A)**, transverse **(B)**, and dorsal **(C)** CT reconstructions depict the left mandibular canine (304), revealing an abnormally wide root canal (Rc) and partial obliteration of the pulp cavity (Pc), characterised by tapered narrowing at its coronal aspect. Additionally, a notable finding is the presence of periapical hypoattenuation (indicated by the purple arrow) at the apex, suggestive of apical periodontitis.

On dental radiographs, variability in pulp chamber and root canal width was observed among teeth with periapical pathology (Figure 3). For example, the root canals of the left mandibular fourth premolar (308) appeared wider than those of the left mandibular third premolar (307) or its counterpart, the right mandibular fourth premolar (408). The left mandibular first molar (309) had significantly narrower root canals and nearly complete obliteration of the pulp chamber compared to its counterpart on the right mandible (409). Similarly, on the occlusal view of the mandible (Figure 3H), the right mandibular canine (404) demonstrated relatively wider root canal space than its counterpart, the left mandibular canine (304), both abnormally wide but at different levels of arrested development (Figures 5, 6). Additionally, the left mandibular canine (304) exhibited partial obliteration of the pulp chamber, characterised by tapered narrowing in its coronal aspect, resulting in a flame-shaped cavity appearance (Figures 4A, 5 6). An inability to visualise the attenuated root canal space was observed in 11 teeth, suggesting near-complete obliteration of the pulp chambers.

External inflammatory resorption was observed at the apices of both roots of the left and right mandibular first molars (309, 409), both roots of the left maxillary third premolar (207), and the distal root of the left maxillary fourth premolar (208).

In addition to the dental radiographic findings, CT imaging revealed abnormally wide pulp cavities in several other teeth: marked abnormal width was observed in the right mandibular first molar (409), while mild abnormal widening was evident in both the right and left maxillary fourth premolars (108, 208) and the left maxillary third premolar (207). Notably, gas was detected within the central portion of the pulp cavity of the right mandibular canine (404), which was evident on the CT images and, to a lesser extent, on the radiographs.

These variabilities in pulp width, in conjunction with periapical lucencies, suggest a sequence of events involving cessation of secondary dentinogenesis, followed by pulp necrosis, occurring at various stages throughout the patient’s lifetime. Furthermore, the CT scan indicated a normal skull appearance and included cervical vertebrae.

### 4.3 Treatment

Full mouth extractions was recommended as every tooth had endodontic disease and apical periodontitis. Treatment was staged over two procedures.

During the first procedure, right intraoral inferior alveolar and maxillary local anaesthetic nerve blocks were performed using 0.7 mL 0.5% bupivacaine^10^ at each site. All teeth in the right maxillary and mandibular quadrants were surgically extracted. All parts of the teeth were very fragile and crumbled easily when force was applied using elevators, making extractions challenging. Complete extraction was confirmed radiographically. Periosteal release of the oral mucosa was performed to allow tension-free closure of the extraction sites, using a simple continuous suture pattern of 4–0 Poliglecaprone 25.^11^

Anaesthetic recovery was uneventful, and the patient was discharged from the hospital the same day. Postoperative oral medications included paracetamol with codeine^12^ (400 mg/9 mg) half a tablet three times daily, meloxicam^13^ (1.5 mg/mL oral suspension) 0.1 mg/kg once daily, and amantadine^14^ (100 mg) 4 mg/kg twice daily. Soft food was recommended for 2 weeks following the procedure. A postoperative examination by the referring veterinarian 14 days post-surgery indicated uneventful healing of the surgical sites. The owner was advised to schedule the patient for second-stage extractions.

The patient returned for the second stage of extractions 11 weeks after the initial surgery, having received daily meloxicam since the first procedure. The same premedication, anaesthesia, and pain relief protocol were administered, including left intraoral inferior alveolar and maxillary dental nerve blocks, using 0.7 mL 0.5% bupivacaine per injection site. The remaining teeth in the left maxillary and mandibular quadrants were extracted. The patient was discharged the same day with meloxicam, paracetamol with codeine and amantadine, as previously described, for pain control. The postoperative instructions and re-examination protocol remained unchanged.

### 4.4 Histopathology and diagnostic interpretation

**Figure 7:**
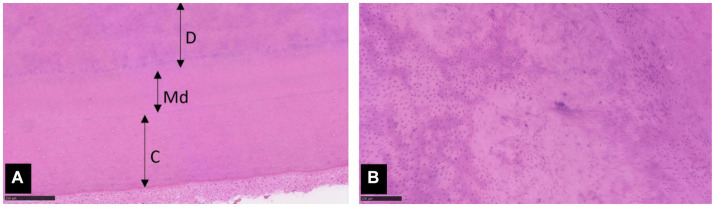
Photomicrographs of extracted tooth 404. **(A)** Dentine **(D)** with mild to moderate disorganisation of tubules borders normal mantle dentine (Md) and cementum **(C)**. Haematoxylin and eosin (H&E) stain, x 100 magnification, scale bar 250 μm. **(B)** Higher magnification of the dentine shows evenly spaced tubules interspersed with areas of tubular disarray. H&E stain, x 200 magnification, scale bar 100 μm.

A previously extracted right mandibular canine tooth (404), kept in buffered formalin, was submitted for histological assessment and histopathologic imaging was reviewed by a pathologist specialised in oral pathology. The samples exhibited diffuse disorganisation of dentinal tubules in both primary and secondary dentine, alongside normal mantle dentine and cementum (Figure 7). Uneven staining of the dentine suggested potential hypo-mineralisation (interglobular mineralisation). Some areas of the tooth had a distinct demarcation line between primary and secondary dentine. The histological features supported a diagnosis of DGI. This tooth had thicker dentine and less severe disorganisation of dentinal tubules compared to other published cases of DGI in dogs (12). These features were attributed to patient age and possible variability in disease severity.

## 5 Diagnosis

Final diagnosis was based on the combined clinical, radiographic, and histopathologic appearance of the patient’s dentition. The absence of systemic disease or pathological long bone fractures, combined with a normal stance and body proportions ruled out the coexistence of osteogenesis imperfecta (OI), making genetic testing for OI unnecessary. Given the radiographic evidence indicating a milder form of DGI, our final diagnosis was DGI-II.

However, there are limiting factors to consider. We lack knowledge about the patient’s pedigree and whether any littermates have developed similar dental symptoms. Furthermore, blood examination was not performed; therefore, abnormalities in phosphorus or calcium levels and their potential influence on the appearance of dentition, although unlikely due to the absence of related symptoms, cannot be completely excluded.

## 6 Discussion

Dentinogenesis imperfecta (DGI) and dentine dysplasia (DD) are the two primary hereditary dentine disorders affecting both human and canine populations. These autosomal dominant genetic disorders are characterised by abnormal dentine structure in primary teeth or both primary and secondary teeth (1). The variable expressivity of these conditions contributes significantly to differences in their clinical expression. General clinical symptoms of DGI and DD include amber, brown/blue, or opalescent brown discolouration of the teeth. Crowns may exhibit a bulbous shape, with variability in pulp chamber width, and roots may appear narrow, short, or exhibit obliterated root canals. Enamel fractures are frequently seen, exposing the softer dentine underneath and resulting in accelerated dental abrasion or attrition (1, 10, 16).

Dentinogenesis imperfecta not associated with osteogenesis imperfecta (OI), is termed non-syndromic and is estimated to affect 1 in 6000–8,000 humans (17). In contrast, dentine dysplasia type I affects approximately 1 in 100,000 individuals in the human population (10). The prevalence of dentine dysplasia type II is currently unknown.

The original, and widely recognised Shield’s classification predates a comprehensive understanding of genetic aetiology. It predominantly relied on clinical and radiographic features, resulting in the delineation of three subtypes of dentinogenesis imperfecta (DGI) – type I, II, and III – as well as two types of dentine dysplasia: type I and II (5).

Where symptoms of DGI type II and III are limited to the dentition, DGI type I (DGI-I) is a syndromic form of DGI associated with osteogenesis imperfecta (OI) a genetic disorder characterised by heightened bone fragility, reduced bone mass, and other symptoms affecting connective tissue (10, 18). In humans, the occurrence of DGI-I in OI exhibits variability (6), with reports indicating its presence in approximately 50% of individuals diagnosed with OI (19).

In humans, OI is genetically heterogeneous, with approximately 90% of cases resulting from mutations of the COL1A1 or COL1A2 genes, which encode procollagen molecules of collagen type I and lead to its quantitative and qualitative abnormalities (6). Collagen type 1 is a protein that plays a major role in building dentinal matrix and is also found in the pulp, bone, and other connective tissues (1, 18, 20). The remaining 10% of OI cases are attributed to recessive mutations in the CRTAP, LEPRE1, PPIB, SERPINH1, FKBP10, PLOD2, SP7, and SERPINF1 genes (6).

Osteogenesis imperfecta has been documented across several canine breeds, such as Collies, Poodles, Norwegian Elkhounds, and Bedlington Terriers (21). In Golden Retrievers and Beagles, OI is associated with mutations in the COL1A1 and COL1A2 genes, respectively (22, 23). Furthermore, mutation in the SERPINH1 gene, responsible for the maturation of type 1 collagen, has been identified as a novel OI gene linked to the manifestation of severe osteopenia and dentinopaenia in two litters of Dachshunds (21, 24).

Teeth affected by DGI-I show marked generalised amber translucent discolouration of deciduous and permanent dentition, bulbous crowns, short constricted roots, and pulp obliteration even before the teeth fully erupt (5, 10). Severity can vary among individuals, with some having complete pulp obliteration and others with normal dentine (10).

In human cases of OI, enamel often exhibits structural changes, particularly with concurrent DGI-I. Primary enamel shows slightly irregular mineralisation, and the dentine-enamel junction (DEJ) may appear straight, smooth, wavy, or normally scalloped. Enamel fractures typically localise at the DEJ and along major incremental lines. These findings, along with observed lower mineralisation and abnormal collagen fibre orientation, likely contribute to enamel shearing (25).

A singular case of OI with DGI-I has been reported in an English Mastiff, attributed to an unidentified genetic mutation. The four-month-old puppy was euthanized due to a poor prognosis after sustaining three long bone fractures within the short time frame. All permanent teeth were undersized and exhibited mild translucency with a pink opalescent hue, with retention of the majority of the deciduous incisors. Incisors had thin dentinal walls. Moreover, the teeth demonstrated fragility and susceptibility to fragmentation under moderate pressure. Histological examination revealed diffuse osteopenia, multifocal odontoblast and ameloblast disorganisation and dysplasia, and dentine hypoplasia suggestive of an OI diagnosis (26).

Dentinogenesis imperfecta type II, known as ‘hereditary opalescent dentine,’ is clinically and radiographically similar to the DGI-I dental phenotype, excluding systemic OI symptoms. However, DGI-II has higher genetic penetrance and more consistent expressivity within families than DGI type I (5, 10). Compared to DGI-I, DGI type II affects both the deciduous and permanent dentition equally, with no normal teeth present. In type II, affected humans within the same family typically exhibit high consistency in the severity, discolouration, and attrition. In contrast, type I shows significant variability in these characteristics among affected family members, and it is not unusual to find normal-appearing teeth in the permanent dentition (5).

Dentinogenesis imperfecta type III, first identified in the Brandywine tri-racial isolate from Maryland and Washington DC (27, 28), exhibits considerable variability in both clinical and radiographic presentations. This variation ranges from pulpal obliteration to a ‘shell teeth’ appearance, characterised by wide pulp chambers and thin dentinal walls, resulting in a hollow appearance of the dentition. Unlike DGI-I and DGI-II, DGI-III commonly presents with multiple pulp exposures in the deciduous dentition (5, 10).

Dentine Dysplasia Type I (DD-I) presents distinctive clinical and radiographic features that differentiate it from DGI. Clinically, the dentition appears to have a normal shape, while radiographically, short roots with sharp conical apical constrictions are evident in both deciduous and permanent dentition. Complete obliteration of the pulp chamber may occur in the deciduous dentition, whereas the permanent dentition typically retains a crescent-shaped remnant of the pulp chamber, positioned parallel with the cemento-enamel junction (5, 10). The dentition is prone to tooth mobility, leading to premature spontaneous exfoliation or loss resulting from minor trauma (29). In contrast to DD type II, DD-I will present with frequent periapical radiolucencies without apparent cause (1, 10). In some cases, dentition may exhibit discolouration, described as slight amber translucency (5), or as brown/blue, yellow/grey, or brown opacity at the incisal level (29).

In the human population, DD-I has been reported to follow an autosomal dominant pattern of inheritance (5). A recent study has identified several pathogenic genes, indicating that DD-I is a genetically heterogeneous disease associated with mutations in the VPS4B, SSUH2, and SMOC2 genes in three different families (11). However, further research is required to elucidate the underlying pathogenic mechanisms. To the authors knowledge, there are no documented cases of DD-I in dogs within the veterinary literature.

In Dentine Dysplasia Type II (DD-II), the deciduous dentition exhibits features resembling those of DGI-II: bulbous crowns with cervical constriction, mild amber translucent discolouration, and pulp obliteration. While the permanent dentition is generally less affected, teeth maintain a normal shape and form, and in most cases, discolouration is not present. However, radiographically, the pulp cavities often display a ‘thistle tube’ shape deformity, with frequent occurrence of pulp stones (5, 10).

In the veterinary literature, there are two documented cases of dentine dysplasia that lack the characteristic tooth root length and shape abnormalities typically associated with DD-I in the human Shields classification system. The first case involves a two-year-old Boerboel dog exhibiting an irregular pattern of mineralisation throughout the entire pulp canal and chamber of five teeth (30). A more diffuse presentation of DD has been described in a four-year-old neutered male terrier mixed breed dog. The permanent dentition exhibited generalised discolouration and diffuse abnormalities in the shape, width, and radio-opacity of the pulp chambers and root canals. All teeth had obliteration or severe narrowing of the coronal pulp cavity, transitioning abruptly to abnormally wide root canals. Despite these dysplastic changes, histopathological examination revealed that the examined teeth remained vital (31).

While these cases adhere to the histopathological criteria for dentine dysplasia, they display distinct characteristics of pulp chamber obliteration that do not align with the Shields classification for either of the two recognised subtypes. Specifically, one case demonstrates generalised pulp chamber obliteration (30), while the other is limited to the coronal portion of the pulp cavity, which is more consistent with the DD-I obliteration pattern (31). This is in contrast to the thistle tube-shaped pulp cavity typical of human DD-II, where the coronal portion remains unaffected and only the root canal is obliterated. Additionally, neither case presents the tooth root abnormalities typical of DD-I, such as shortened or absent roots. As such, both cases fulfil the broader criteria for dentine dysplasia but cannot be accurately classified as either DD-I or DD-II according to the human Shields classification.

Dentine dysplasia type II and the two non-syndromic subtypes of dentinogenesis imperfecta, type II and III, are caused by variable types of mutations at different locations of the DSPP gene (7, 8), which implies possible allelic nature of those disorders (8, 9, 32). Moreover, several studies have found considerable phenotypic variation among individuals with DSPP mutations (9, 32). Therefore, this suggests that DD-II and DGI-II represent different severities of the same underlying genetic condition rather than distinct diseases in humans.

The DSPP gene, located on human chromosome 4, consists of four introns and five exons, with the first exon being non-coding. Exons 2–5 encode a single transcript that is cleaved into three major proteins, dentine sialoprotein (DSP), dentine phosphoprotein (DPP) and dentine glycoprotein (DGP), all of which are uniquely expressed in odontoblasts. DSP is encoded within exons 2, 3, 4 and the beginning of exon 5, while DGP and DPP are encoded by the end of exon 5. These non-collagenous proteins are essential parts of the dentine extracellular matrix (DECM), with DSP constituting 5–8% and DPP being the predominant non-collagenous protein, making up to 50% of the DECM (8, 10, 29, 33–35). All three proteins undergo multiple post-translational modifications, such as glycosylation and phosphorylation, which facilitate and regulate the mineralisation of dentine (29).

Experimental DSPP-null mice developed tooth defects such as enlarged pulp cavities, widened predentine zone, decreased dentine width, and a high incidence of pulp exposures resembling the human DGI-III phenotype (36) confirming the crucial role of DSPP in the dentine mineralisation process.

Dentine phosphoprotein plays a role in binding large amounts of calcium and its bridging, but also in hydroxyapatite nucleation, regulating the size and shape of the hydroxyapatite (7, 34). It is believed to be involved in the maturation of mineralised dentine (37).

The role of DSP in dentine mineralisation remains unclear. Nonetheless, it is thought to play a part in initiating the dentine mineralisation (7, 37).

Dentine glycoprotein has been isolated from human, pig, rat, and mouse. Porcine DGP shares 81% amino acid identity with human DGP, 49% with rat, and 47% with mouse. Porcine DGP consists of 81 amino acids, with 12 negatively charged (Asp., Glu) and 12 positively charged (Arg, Lys) residues, and its most abundant amino acids are glycine and serine. DGP undergoes post-translational modifications, including four phosphorylated serine residues and one N-linked glycosylated asparagine, which enhance its affinity for hydroxyapatite. The sequence of DGP shows high conservation among mammals, particularly regarding length and post-translational modifications. These modifications make DGP acidic, a property believed to facilitate its binding to dentine crystals and maintain its association with the mineralised dentine matrix (35).

Based on the DSPP gene sequence, mutations can be categorised into three groups: those occurring in the coding sequence of (1) the signal peptide, (2) DSP, or (3) DPP. It is proposed that a missense mutation in the signal peptide impairs or stops the translocation of DSPP into the endoplasmic reticulum (ER), which prevents intense post-translational modifications reducing its availability and function, which consequently leads to reduced secretion of DSP and DPP into the extracellular matrix affecting dentine formation. Mutations in the DSP coding sequence and the first three amino acids typically result in DGI-II. Nonsense mutations in exon 3 can lead to the premature termination of transcription, incomplete translation and truncated DSPP formation where only DSP is formed. Mutations resulting in skipping of the entire exon 3 manifested severe phenotype of DGI-II without hearing loss. It is unclear whether mutations in the DSP sequence exclusively disrupt DSP formation or also affect DPP protein (7).

Mutations in exon 5 of the DSPP gene, encoding the entire DPP, show the most diverse range of phenotypes: DD-II, DGI-II, and DGI-III. These mutations, often deletions and/or insertions, lead to frameshifts (7).

The majority of mutations in the DSPP gene result in phenotypes of DGI-II and/or DGI-III or DD-II. However, among the over 30 identified mutations in the DSPP gene, some do not lead to dental phenotypes (7).

### 6.1 Primary dentine disorders classification issues

Since the establishment of Shields’ classification in 1973, several authors have argued that classifications based solely on phenotypic characterisation inadequately capture the complexity of genetic dentine disorders. Shields’ classification aimed to group inherited dentinal disorders by their phenotypes, with the hope that pathological features would fit into single categories with common genetic causes (10). However, these disorders exhibit variable genetic aetiologies and overlapping clinical characteristics that defy strict categorisation into subtypes (1, 10, 29). Features such as the bulbous crowns and cervical constriction characteristic of DGI-II, the thistle-tube pulp chambers typical of DD-II, and the wide pulp chambers with multiple pulp exposures associated with DGI-III are not exclusive to these subtypes. Different dental phenotypes can also be observed within affected individuals from the same family, and there is significantly variable severity within these subtypes (10).

In response to these complexities, the Online Mendelian Inheritance in Man (OMIM) database^15^ provides up-to-date, detailed, and curated information on molecular genetic variations and their relationships to human diseases. According to OMIM, DGI-I has been reclassified as OI (OMIM #166200) due to their genetic overlap. Consequently, the dental phenotype known as DGI-II is now classified as DGI-I (OMIM #125490). DGI-III and both types of dentine dysplasia (DD-I and DD-II) have retained their original names from Shields’ classification (38).

In order to avoid confusion, we have used the original Shield’s classification throughout this manuscript (5), as it remains the most widely recognised, instead of the OMIM re-classification.

The Brandywine isolate, which serves as the prototype for DGI-III, is caused by the 5′-DSPP mutation c.49C > T; p.Pro17Ser. Additionally, other missense mutations affecting the same DSPP amino acid have been found to cause symptoms similar to those associated with DGI-III. Based on studies with genetically modified mice, it is observed that 5′-DSPP mutations affect protein trafficking and secretion, inducing both dentine and enamel defects, leading to DGI-III, as seen in the Brandywine isolate. In contrast, 3′-DSPP −1 frameshift mutations primarily cause dentine defects through severe cell toxicity in odontoblasts, resulting in DGI-II or DD-II phenotypes. Both types of mutations result in autosomal dominant inherited dentine defects (14).

To further refine classification considering these findings, the recently proposed modified Shields classification emphasises the importance of obtaining a diagnosis of inherited dentine disorders based on genetic aetiology rather than solely on clinical features. Specifically, it suggests that a diagnosis of DGI-II, a milder form of non-syndromic DGI, should be applied to patients with 3′-DSPP −1 frameshift mutations. Conversely, those with dominant 5′-DSPP mutations, except those in the signal peptide coding segment, should receive a DGI-III diagnosis (14).

However, certain 5′-DSPP defects, such as the c.52-6 T > G splice junction mutation, can lead to a milder DD-II phenotype by allowing the production of some normal DSPP protein. This milder DD-II phenotype is due to a dose effect, with fewer defective DSPP proteins synthesised compared to other mutations. While not all 5′-DSPP defects cause severe dental malformations, only these defects directly affect amelogenesis, leading to rapid dental attrition as seen in DGI-III. Transient synthesis of the 5′-DSPP mutated protein can result in pathology that interferes with the ability of ameloblasts to produce fully normal enamel. Classification based on genetic aetiology is further complicated by the fact that 5′-DSPP and 3′-DSPP mutations exhibit variations in the severity of the clinical phenotype (14).

### 6.2 Histopathology in DGI diagnosis

Common features for all heritable dentine disorders in Shields’ classification are the presence of normal enamel and mantle dentine (5).

In DGI, normal mantle dentine meets with dysplastic primary and secondary dentine. In humans, the abnormal dentine often hypertrophies into the pulp chamber and pulp canal. Over time, irregular dentine replaces the pulpal space to be nearly or fully obliterated. Abnormal dentine can be atubular or have fewer dentinal tubules that exhibit variable size and direction. Additionally, interglobular calcification can be found within the abnormal dentine, and pulp stones may also be present within the pulp chamber (5, 38, 39). These histological features are similar to reported cases of DGI in dogs (12, 15, 31). However, over-production of dentine and pulp canal obliteration has not been described in dogs with DGI. This might be explained by the young age of dogs previously reported. It is also possible that pulp canal obliteration may be a secondary change related to severe attrition of teeth affected by DGI. Histological features of teeth from individuals with osteogenesis imperfecta (OI) are indistinguishable from those of DGI, making differentiation based only on these features impossible (15).

In dentine dysplasia, the layer of mantle dentine and most of the primary and secondary dentine are histologically normal. However, deeper layers of secondary dentine may by exhibit amorphous and atubular regions. The deposition of secondary dentine may be exaggerated and lead to pulp canal obliteration within the crown or root in teeth affected by DD-I and DD-II, respectively. Globular masses of irregular dentine may form stones that also occupy pulp space (5, 39). Unlike DGI, pulp canal obliteration in DD seems to be related to the primary abnormality in dentinogenesis rather than secondary to attrition.

### 6.3 Differential diagnosis

During initial clinical evaluation, DGI may mistakenly be classified as a primary enamel disorder, given the shared symptoms such as enamel loss, exposure of the underlying dentine, and dental discolouration. Genetically inherited forms of enamel dysplasia are known as amelogenesis imperfecta (AI). Severe physiological changes during formation of the tooth bud can disrupt enamel formation due to the sensitivity of ameloblasts to their environment (20). The location and appearance of enamel defects are significantly influenced by the stage of enamel formation during which damage to the ameloblasts occurs. While the underlying cause of such damage seems less significant, various internal and external factors can lead to similar clinical defects (38).

Common systemic causes, such as toxin exposure, infections (systemic and localised), periods of malnutrition, metabolic disorders, and maxillofacial trauma, typically result in localised, focal defects rather than diffuse changes (15), characterised by distinctive bands of malformed enamel (15, 20). Canine distemper virus in puppies that survive the infection during tooth development will result in irregular, patchy, or annular enamel defects (15, 40). Tetracycline-induced disturbances in teeth can lead to defects, characterised by brown pigmentation bands or complete pigmentation within the enamel, along with hypoplasia or enamel absence. The extent of damage is dependent on the dosage and duration of the antibiotic therapy (20).

Amelogenesis imperfecta affects both the deciduous and permanent dentition and results from mutations in genes responsible for encoding enamel proteins or proteases. Human classification models categorise AI into three phenotypic subtypes—hypoplastic, hypomature, and hypocalcified—based on the stage at which the amelogenesis is disrupted (15, 20).

Although (44) first identified familial-type enamel hypoplasia in Standard Poodles in Sweden, resembling amelogenesis imperfecta (AI), the precise genetic mutation responsible for this condition has not yet been explored in the veterinary literature. Breed-specific genetic mutations resulting in AI have been identified in Italian Greyhounds (ENAM gene), Samoyeds (SCL24A4), Parson Russell Terriers (ENAM and ACP4), Akita and American Akita (ACP4) with a carrier frequency of between 1 and 22% (41–43).

Clinical manifestations of amelogenesis imperfecta (AI) include discolouration of the permanent dentition with variably affected deciduous dentition. The discolouration can be limited to a few teeth or generalised, with discrete signs that are difficult to recognise in deciduous teeth and more visible as dark brown blotches in the permanent dentition where the enamel is thin or absent (44).

Affected permanent teeth in dogs may appear small and pointed, with wider interdental spaces. Unlike in humans, tooth fractures are not observed in dogs with this condition (41). The roughened tooth surfaces predispose many individuals to periodontal disease due to their plaque-retentive nature. Akitas and Samoyeds show a higher predilection for severe dental abrasion (42, 43), which can mimic the clinical symptoms of DGI-II, leading to potential diagnostic challenges.

Genetic testing for AI in dogs^16^ is currently limited to the mutations that have been identified, including ENAM (both variations), ACP4, and SCL24A4 genes. This testing can be particularly useful in breeds predisposed to AI, such as Italian Greyhounds, Samoyeds, Akitas, and American Akitas, when making a diagnosis. The list of mutations associated with AI in dogs has not yet reached the extensive array identified in human genetic studies of the disorder. Consequently, distinguishing AI from other enamel disorders, such as non-syndromic DGI, can be challenging when relying solely on an examination of the oral cavity in the conscious patient, especially in the presence of similar clinical symptoms.

Histopathological analysis plays a pivotal role in providing a conclusive diagnosis in DGI cases, as it reveals abnormal arrangements of dentinal tubules while maintaining normal cementum and enamel. In contrast, individuals with AI exhibit significant variability in the typical prism structure of enamel, which can appear highly disorganised (15). It is essential to note that routine tooth histopathological examination tends to remove almost all of the enamel in decalcified sections due to its predominantly inorganic composition. To accurately analyse the histopathological characteristics of enamel, costly and time-consuming non-decalcified sections are often required, which are typically only available from specialised laboratories (15).

Furthermore, if an enamel disorder is suspected, the extraction of an intact tooth solely for the purpose of diagnostic histopathological examination may not be justified. However, in the presence of severe clinical symptoms, abnormal, non-functional, or less important teeth may be chosen for extraction for prognostic purposes.

In cases of DGI, affected teeth commonly exhibit endodontic disease, fractures, or exposed pulp, often necessitating extraction and providing samples for histopathological examination. Prognostic assessment is important if non-syndromic DGI is suspected, given the likelihood of development of pan-oral endodontic disease.

In contrast, dogs affected by AI have a much better prognosis and tend to retain their teeth throughout their lifetime, indicating that the hardness and strength of their teeth are not significantly compromised (15, 41). However, these dogs may be more susceptible to plaque, calculus, and progressive periodontal disease (42). Therefore, histopathological examination is beneficial for prognostic assessment in both conditions.

Radiographic examination of the dentition serves as a valuable, relatively low-cost diagnostic tool to identify pathological changes associated with non-syndromic DGI or their absence. Teeth affected by amelogenesis imperfecta do not exhibit pulp chamber obliteration and periapical pathology is less common (1). When the radiographic findings are considered in combination with the clinical appearance of the teeth, AI or other enamel disorders should be distinguishable from DGI.

Regional odontodysplasia (RO) is a rare, localised, nonhereditary developmental disorder of teeth that significantly impacts the formation of enamel, dentine, and pulp. This condition can occur in both deciduous and permanent teeth. Most cases are suspected to be idiopathic; however, some have been linked to various syndromes, growth defects, neurological disorders, and vascular malformations, such as vascular nevi of the head and neck (38).

The disorder can be distinguished from DGI as it typically affects a localised or focal area of the dentition, involving several adjacent teeth, rather than the entire dentition. RO most commonly affects the maxillary teeth. Within the affected region, unaffected teeth may be interspersed among altered teeth (38).

Radiographically, the affected teeth display very thin layers of enamel and dentine surrounding an enlarged, radiolucent pulp chamber, which gives the teeth a characteristic “ghost teeth” or “fuzzy” appearance. A marked lack of contrast between the dentine and enamel often renders them indistinguishable. The affected teeth may have short roots and open apices, with enlarged pulps frequently containing pulp stones and denticles. However, mature teeth tend to have more normal-looking canals and well-developed apices. The crowns of these teeth may appear small, irregular, and discoloured, ranging from yellow to brown, with rough, pitted surfaces (38, 45).

Histologically, the teeth are characterised by variable enamel hypoplasia and hypocalcification. In some areas, the dentinal tubules may be reduced in number, irregularly distributed, and abnormally oriented. Features such as invaginations extending from the enamel surface to the dentine, dentinal clefts, diffuse calcification of the pulpal tissue, and pulpal stones are also observed (45).

Delayed or failed tooth eruptions, as well as early tooth exfoliation, are common. Periapical inflammatory lesions are also frequent because of teeth abnormalities which expose the endodontic system to the oral cavity (38, 45).

Only a single case of RO has been reported in veterinary literature in the right maxillary canine of a 7-month-old Beagle with no history of facial trauma (46).

Both DGI and RO can result in abnormal dentine formation. However, they differ significantly in their distribution, enamel presentation, and number of teeth affected. RO has a localised, irregular effect on both enamel and dentine where morphologically normal teeth can be present among affected dentition. DGI has a generalised, uniform impact on dentine formation in all teeth, with histopathologically normal enamel being lost from occlusal surfaces secondarily due to mechanical damage or abrasion.

The dental symptoms of Dental-Skeletal-Retinal-Anomaly (DSRA), a newly described collagenopathy in Cane Corso dogs, mimic the DGI dental phenotype (47). Both the deciduous and permanent dentition or only permanent dentition are brittle and translucent, exhibiting brown or pink opalescent discolouration and varying degrees of multifocal enamel loss. Delayed dentinal deposition leads to thin dentinal walls and shell-like teeth appearance as in DGI-III. The teeth are prone to crown/root fractures as well as the development of endodontic disease. Histopathological findings align with a DGI diagnosis. However, affected dogs also exhibit additional symptoms, including shortened stature, a history of lameness, and progressive vision loss due to progressive retinal atrophy (PRA) (48, 49). This condition is inherited in a monogenic autosomal recessive manner, with a mutation in the MIA3 gene identified as the cause. The MIA3 gene encodes the endoplasmic reticulum (ER) membrane protein TANGO1, which is suspected to play a crucial role in the exportation of large proteins like collagens across the ER membrane. Dysfunction in this protein leads to a generalised decrease in collagen, resulting in multi-organ effects (47, 48). Suspected DSRA individuals can be tested for the MIA3 gene mutation.

In Border Collies, a missense variant (c.899C > T, p.A300V) in the FAM20C gene, inherited in a recessive manner, has been identified as the cause of dental hypomineralisation, leading to severe dental abrasion. Affected dogs present with light brown enamel discolouration, reduced crown height, variable severity of pulp cavity obliteration, pulp exposure in multiple teeth, and occlusal enamel loss, all of which share clinical similarities with DGI. Histopathological examination revealed hypoplastic enamel, with primary dentine showing a normal tubular pattern, however, the middle zone was distinctly globular, and the central zone adjacent to pulp chamber exhibited a slightly irregular tubular pattern (50). In humans, mutations in the FAM20C gene are responsible for highly lethal Raine syndrome, a neonatal osteosclerotic bone dysplasia (OMIM #259775), characterised by craniofacial anomalies, osteosclerosis and variable hypophosphataemia leading to dental and bone hypomineralisation. However, the condition observed in Border Collies is primarily limited to dental symptoms, without the accompanying hypophosphataemia and craniofacial anomalies characteristic of Raine syndrome (50). The abnormalities in bone and tooth development observed in both Border Collies and humans with FAM20C gene mutations can be attributed to the gene’s role in promoting the differentiation of osteoblast lineages and indirectly regulating phosphate homeostasis through the mediation of FGF23, a hormone known to promote phosphate excretion (50, 51). Genetic testing for FAM20C gene is widely available.

**Figure 8:**
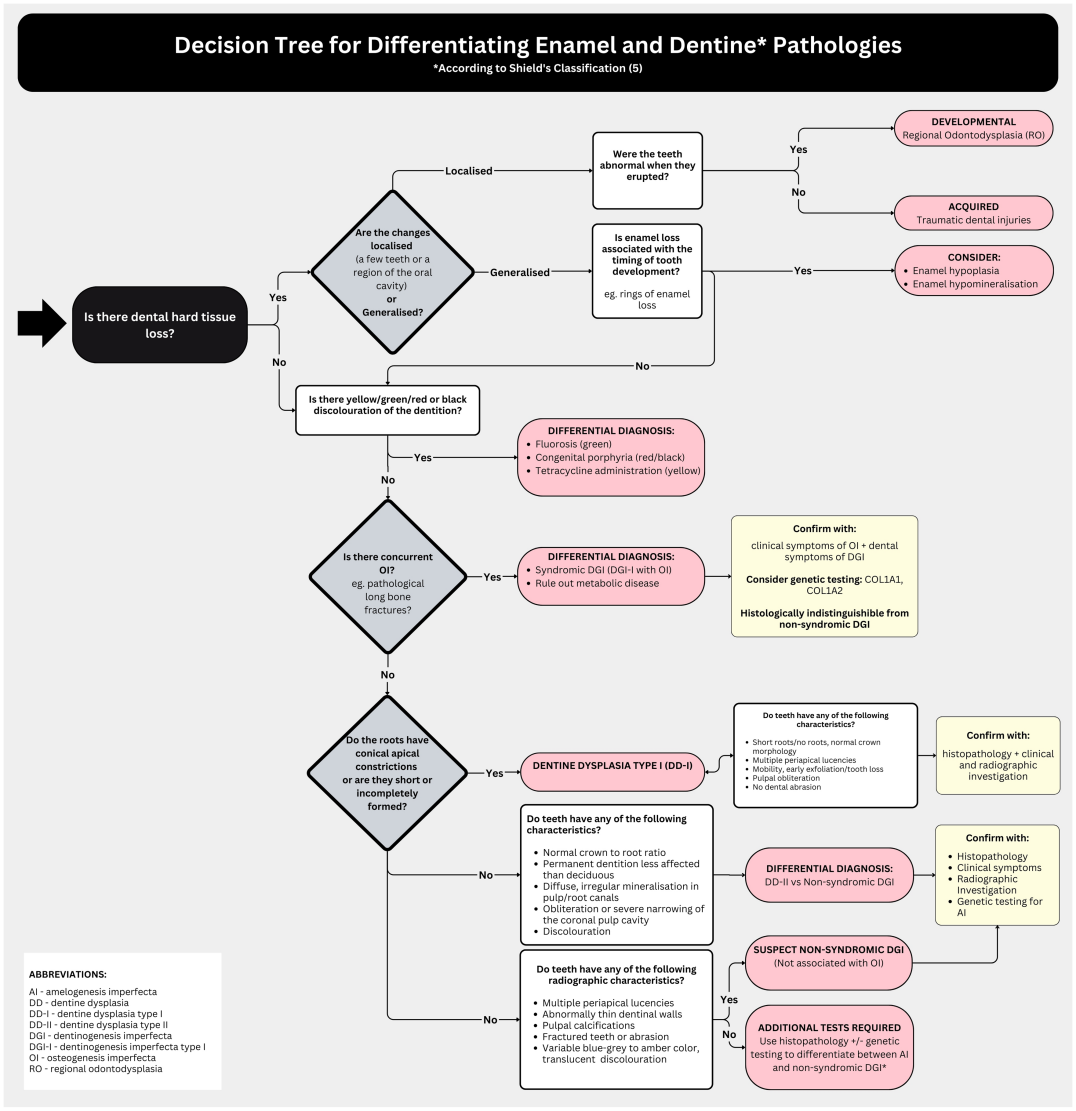
Decision tree for differentiating enamel and dentine pathologies.

To facilitate clinical decision-making regarding enamel and dentine disorders, the provided decision tree serves as a valuable tool (Figure 8).

### 6.4 Diagnosis and comparison of documented non-syndromic DGI cases in dogs

Diagnosis of non-syndromic DGI in dogs has been based on phenotypic similarity to human DGI-II and the absence of OI symptoms: a one-year-old Labrador Retriever, a one-year-old mixed breed dog, and a 23-week-old Doberman Pinscher (12, 13). The first case was initially classified as DGI-III due to the severity of the symptoms. Progressive narrowing of the pulp cavity on serial radiographs suggested a similarity to Shields’ DGI-II (13). Only one of these documented cases has been tested for known genetic mutations associated with OI as part of the diagnostic process (12). Further investigation should be considered for dogs used in breeding or when the final diagnosis is in doubt.

Common symptoms of non-syndromic DGI in dogs were consistent with those observed in human patients. These were characterised by typical discolouration, accelerated attrition, enamel loss, tooth fragility, crown and root fractures, and multiple periapical lucencies, even in teeth without evident pulp exposure. The severity varied from mild to severe, with the most severe case showing multiple pulp exposures in permanent teeth at an early age and our case being the mildest. Shell teeth appearance was also noted, with subsequent radiographs showing dentine thickening over time, although thinner than expected developmentally. Interestingly, two dogs were presented with traumatic malocclusion as a chief complaint (12, 13). A detailed comparison of all documented cases in veterinary literature is presented in Table 1.

**Table 1:** Comparison summary of the recently reported canine dentinogenesis imperfecta type II.

	Labrador retriever current case	Mixed breed (12)	Labrador retriever (13)	Doberman Pinscher (12)
Age at presentation	6 years 4 months	1 year 1 month	1 year	<1 month*
Sex	MN	FN	FE	ME
Chief complaint	Severe generalised dental abrasion Generalised discolouration Oral pain	Severe, generalised abrasion discolouration Assessment of missing dentition	generalised permanent dentition discolouration malocclusion	traumatic occlusion skeletal malocclusion
Persistent deciduous dentition	Yes	No	No	Yes (607 and 706)
Malformed dentition	No	The right maxillary fourth premolar (108) malformed and missing approximately half of the clinical crown	No	No
Dentition affected	Deciduous and permanent	Permanent Deciduous—Unknown	Deciduous and permanent	Deciduous and permanent
Fractures	Multiple Incisors at the level of CEJ Deciduous tooth fracture	Multiple Incisors At the level of CEJ Root fractures	Multiple root fractures	Present
Dental abrasion	Severe generalised abrasion	Severe generalised abrasion at 13 months	Significant generalised Incisors mandibular premolars	Occlusal surfaces of mandibular molars, incisive surface of the incisors at 15 months
Enamel loss	Severe occlusal surfaces	Severe generalised	Mild	Mild occlusal surfaces
Periapical pathology First Noted	All dentition even teeth without pulp exposure 6 years	Multiple teeth 13 months	Multiple (including teeth without pulp exposure) 12 months	Multiple teeth, including teeth without pulp exposure 14 months
Pulp exposure	Yes	Yes multiple	Yes, multiple	Yes
Generalised discolouration of the dentition	Deciduous—translucent Permanent—Amber brown	Deciduous Unknown Permanent—golden-brown	Deciduous—purple Permanent—blue-purple /blue-grey	Deciduous—abnormal Permanent—blue-grey
Initial Pulp chamber width of permanent dentition	Generalised variable pulp chamber widths. Obliterated pulp chambers Irregular secondary dentine deposition in pulp chamber Obliterated root canals Pulp chamber progressive maturation present	Variable pulp chamber widths Wide pulp chambers abruptly tapering to a thin width between the middle and apical third of the roots. Complete obliteration of the apical root canal with wide pulp chamber within the coronal third of the root.	Wide pulp chambers and thin dentinal walls in relation to normal at this age Pulp chamber progressive maturation present but slow	Variable pulp chamber widths Initially abnormally wide pulp chambers Shell like appearance of teeth
Pulpal calcifications	Possible dystrophic calcification	No	No	Possible pulp stone**
Root canal obliteration	Yes	Yes	?	?
Secondary dentine deposition	Varied between teeth	Minimal	Thin dentinal walls, Delayed deposition	Delayed, irregular in radicular portion of the tooth
Pulp chamber obliteration	Complete obliteration of the pulp chamber and root canal	Near-complete obliteration of the pulp chambers for the entire tooth length	No	No
Malocclusion	Normocclusion	No	Class 3 with linguoversion of the right mandibular canine (404)	Class 4; Rostrocaudal asymmetric skeletal malocclusion Rostral crossbite Left mandibular canine linguoversion
Traumatic occlusion	No	No	Yes	Yes
Delayed exfoliation of deciduous dentition	Yes	Unknown	No	Yes
Other puppies affected in the litter	Unknown	Unknown	No	No
Genetic testing	No	Negative—Test for OI COL1A1, COL1A2, and SERPINH1 known gene mutations	No	No
Treatment	Staged full mouth extractions	Selective extraction therapy	Staged full mouth extractions	Selective extraction therapy
Long term follow up	Yes	No	No	No
Diagnosis***	DGI-II	DGI-II	DGI-II, initially DGI- III	DGI-II

*23 weeks. **Small round density mid coronal pulp chamber of (104). ***According to Shield’s Classification (5). FN, female neutered; FE, female entire; MN, male neutered; ME, male entire; DGI-II, dentinogenesis imperfecta type II; DGI-III, dentinogenesis imperfecta type III.

### 6.5 Treatment literature review

In human dentistry, dental treatment strategies focus on preserving the compromised dentition by protecting the teeth against wear and restoring them in accordance with the specific structural properties of the defective dentine (52). This can include adhesive dentistry, periodontal surgery, implant-supported prostheses, orthodontic treatment, and orthognathic surgery (53).

Early diagnosis and adherence to recommended treatments can achieve good aesthetics and function (1). Strict oral hygiene, dietary instructions, topical fluoride varnish, and frequent follow-ups are essential preventive measures (54). Early tooth loss and root or crown fractures can be anticipated (52). Radiographic monitoring is also recommended due to frequent spontaneous apical periodontitis formation in DGI-II (1).

In paediatric human patients, treatment aims to preserve the vitality, structure, and size of the dentition, minimise nutritional deficiencies and allow for normal facial bone growth (54, 55), prevent possible temporo-mandibular joint (TMJ) problems. DGI-affected teeth exhibit excessive decay, and the possibility of optimal treatment decreases with patient age (55).

For deciduous and young permanent posterior teeth in human patients, the placement of stainless steel crowns (SSCs) is recommended as soon as they erupt to prevent excessive loss of tooth structure (55). When placing SSCs on deciduous teeth, a pulpotomy with mineral trioxide aggregate may need to be performed prior to crown placement. However, due to pulp obliteration, which is common in human DGI-II and has been observed in the permanent dentition in our case, this treatment can be challenging and may potentially worsen the prognosis for a successful outcome (55).

Covering permanent human molars with SSC early on is crucial to prevent wear, ensure proper occlusal relationships, and preserve tooth vitality (56). A recently published case report describes a two-stage rehabilitation approach using CAD/CAM technology for crown preparations, allowing for minimally invasive procedures. This method utilised titanium alloy to crown the posterior teeth and resin composite to restore the anterior teeth. A three-year follow-up demonstrated stable treatment outcomes, effectively preventing further dental wear (57). However, enamel fractures and detachment can occur following cementation due to abnormal DEJ in human DGI-II (2, 58).

Dentine and enamel form a strong bond at the dentine-enamel junction (DEJ). Normal scalloping of the DEJ is commonly observed in both types of dentine dysplasia and has been noted with variable frequency and severity in all three subtypes of DGI in humans (5).

Although enamel in DGI-II is typically reported to be normal and is thought to be easily exfoliated due to an abnormal dentino-enamel junction, two studies identified a specific DSPP mutation associated with DGI-II that also affects early-stage amelogenesis. This mutation results in unique hypoplastic enamel defects localised to the occlusal third of the crown (3, 4). The precise mechanism by which the DSPP mutation influences amelogenesis remains unclear. However, it is hypothesised that the mutation causes disturbances in ameloblast function during or shortly after DSPP expression in the preameloblast stage, leading to residual ameloblast pathology that affects enamel formation (3).

Teeth affected by DGI-II exhibited significantly altered physical properties compared to healthy teeth. The Vickers hardness of DGI-II teeth was seven times lower, and their Young’s modulus was six times lower than that of healthy teeth (59). Additionally, mineral composition analysis revealed a substantial decrease in calcium ions, an increase in phosphorus content, and a reduced calcium-to-phosphorus ratio in DGI-II-affected teeth (59, 60).

These structural defects and altered mineral content are suspected of contributing to high attrition rates, increased tooth fragility, and challenges in achieving durable dental restorations in DGI-II patients (2, 59, 61).

Traditional restorative materials, designed based on the properties of healthy teeth, may not perform adequately for patients with DGI-II due to the significant differences in enamel and dentine properties. The reduced hardness, elasticity, and stiffness lead to micromovement and potential loss of retention for restorations (59).

It is speculated that in cases where normal enamel exists, exposing the prismatic enamel may enhance mechanical retention (2). Minimal tooth preparation is suggested to remove only 30 microns of the aprismatic enamel layer to enable effective bonding (62). A systematic review from 2021 on dentine disorders and adhesive treatments shows that bonding to enamel offers a better prognosis and worsens when dentine is exposed (62). Furthermore, using glass ionomer cement (GIC) and dentinal adhesives as the first layer, with composite restoration on top, may improve the durability of the restoration by creating a chemical bond (63). GIC is favoured for cementing because of its fluoride-releasing and recharging capabilities and enhanced adhesion properties, which are particularly advantageous in cases of severe hypomineralisation (56). The choice of adhesive system can significantly impact the clinical outcome of resin restoration adhesion to hypomineralised enamel, which generally exhibits lower shear bond strength compared to normal enamel. Self-etching adhesives are suggested to offer better adhesion in such cases due to their simplicity, hydrophilicity, and reduced enamel loss compared to phosphoric acid etching (56).

Endodontic treatment in patients with DGI-II has a poor prognosis due to the considerable challenges presented by abnormal pulp morphology, which often renders standard procedures unfeasible because of pulp chamber obliteration (52) and sclerosed root canals (64). Most endodontically treated teeth in DGI patients are eventually extracted. It is advisable to perform endodontic treatment while the patient is still young and the canals are accessible. Although successful endodontic treatment is possible (64, 65) treated teeth should be extensively monitored for potential treatment failure (52). Teeth that have undergone endodontic treatment should subsequently be protected with full metal jacket crowns (64, 65).

## 7 Conclusion

Translating the treatment strategies for DGI-II from human dentistry to veterinary medicine necessitates adapting principles and techniques to accommodate the anatomical and physiological differences in animals. As in humans, early diagnosis is crucial for achieving a favourable prognosis, given the varying severity of non-syndromic DGI documented in dogs to date. Selective extractions or staged full-mouth extractions have often been the treatment of choice due to the severity of clinical cases at presentation and the poor prognosis for retaining dentition longer term (12, 13).

In dogs, the deciduous dentition undergoes rapid exfoliation and is present for a comparatively brief duration, rendering restorative or prosthodontic treatment impractical (13). Nevertheless, early intervention with extractions should be undertaken in cases of crown fractures with pulp exposure, signs of mobility, infection, or pain before complete exfoliation of deciduous dentition. Adhering to good oral hygiene practices at home, implementing dietary recommendations such as avoiding hard food and chews, including potentially abrasive toys, applying topical fluoride varnish during neutering procedures, and conducting regular follow-up appointments are essential preventive strategies.

For permanent dentition, a human paediatric two-stage approach could be employed, involving indirect complete restoration using titanium alloy crowns for functionally important teeth such as canines, the maxillary fourth premolars, and mandibular first molars (57). The use of stainless steel or titanium alloy crowns requires minimal crown preparation compared to ceramic options, making them preferable for the thin-walled and wide pulp chambers of DGI affected teeth, where significant difficulties in tooth preparation are anticipated. Temporary prosthodontic crowns should be placed to prevent further enamel loss before the final restoration can be completed.

Early prosthodontic treatment for permanent teeth enhances the likelihood of prosthodontic success due to a lesser degree of abrasion, a better crown-to-root ratio, and a higher amount of residual enamel. Adhesive materials form stronger bonds with enamel than with exposed dentine, which is advantageous in early interventions.

Endodontic treatment is feasible; however, it presents challenges due to potential pulp chamber obliteration or root canal sclerosis, leading to a generally poor prognosis. In those situations, extractions or frequent follow-up radiographic examinations should be considered.

Veterinary practitioners must consider the specific needs and compliance of both animal patients and their owners, ensuring treatments are effective and practical within a veterinary context. Regular follow-ups and radiographic monitoring are essential for early detection and management of complications, thereby enhancing the overall success of the treatment plan for dogs with non-syndromic DGI. Efforts to preserve teeth in these patients are likely to necessitate multiple general anaesthetics and incur high overall costs. Therefore, good patient selection and consideration of overall clinical health are crucial in treatment decision-making.

## Data Availability

The original contributions presented in the study are included in the article/supplementary material, further inquiries can be directed to the corresponding author.

## Footnotes

^1^Laboklin, UK.

^2^Sedator® 1.0 mg/mL, Dechra Veterinary Products Limited, Northwich, Cheshire, UK.

^3^Comfortan® 10 mg/mL, Dechra Veterinary Products Limited, Northwich, Cheshire, UK.

^4^Propofol®-Lipuro Vet 10 mg/mL emulsion, Virbac, Carros, France; Virbac Ltd., Woolpit, Suffolk, UK.

^5^IsoFlo® (isoflurane) 100 mL, Zoetis UK Ltd., Leatherhead, Surrey, UK.

^6^Acp 2 mg/mL, Elanco Europe Ltd., Hook, UK.

^7^Paracetamol 10 mg/mL 100 mL Solution for Infusion, B.Braun Medical, Sheffield, South Yorkshire, UK.

^8^Metacam® 5 mg/mL, Boehringer Ingelheim Animal Health UK Ltd., Bracknell, Berkshire, UK.

^9^Anesketin 100 mg/mL, Dechra Veterinary Products Limited, Northwich, Cheshire, UK.

^10^Bupivacaine Hydrochloride 0.5% w/v solution for injection, ADVANZ Pharma, UK.

^11^Monocryl®, Poliglecaprone 25, 4:0 sutures, Ethicon (Johnson & Johnson), UK.

^12^Pardale-V Oral Tablets, Dechra Veterinary Products Limited, Northwich, Cheshire, UK.

^13^Metacam® 1.5 mg/mL Oral Suspension for Dogs Boehringer Ingelheim Animal Health UK Ltd., Bracknell, Berkshire, UK.

^14^Amantadine Hydrochloride 100 mg capsules, Teva UK Ltd., UK.

^15^https://www.omim.org—Online Mendelian Inheritance in Man (OMIM).

^16^Laboklin, UK.
